# Short postsurgical antibiotic therapy for spinal infections: protocol of prospective, randomized, unblinded, noninferiority trials (SASI trials)

**DOI:** 10.1186/s13063-020-4047-3

**Published:** 2020-02-06

**Authors:** Michael Betz, Ilker Uçkay, Regula Schüpbach, Tanja Gröber, Sander M. Botter, Jan Burkhard, Dominique Holy, Yvonne Achermann, Mazda Farshad

**Affiliations:** 1 Department of Orthopedic Surgery, Balgrist University Hospital, Zurich, Switzerland; 2 University Spine Center Zürich, Balgrist University Hospital, Zurich, Switzerland; 3 Unit for Clinical and Applied Research, Balgrist University Hospital, Zurich, Switzerland; 4 Infectiology, Balgrist University Hospital, Zurich, Switzerland; 5 Internal Medicine, Balgrist University Hospital, Forchstrasse 340, 8008 Zurich, Switzerland; 6 Swiss Center for Muskuloskeletal Banking, Balgrist Campus AG, Zurich, Switzerland; 7 Division of Infectious Diseases and Hospital Epidemiology, University Hospital Zurich, Zurich, Switzerland

**Keywords:** Spinal infection, Osteomyelitis, Spondylodesis, Antibiotic duration, Remission, Failure, Financial cost, Adverse event

## Abstract

**Background:**

There are several open scientific questions regarding the optimal antibiotic treatment of spinal infections (SIs) with or without an implant. The duration of postsurgical antibiotic therapy is debated.

**Methods:**

We will perform two unblinded randomized controlled trials (RCTs). We hypothesize that shorter durations of systemic antibiotic therapy after surgery for SI are noninferior (10% margin, 80% power, α = 5%) to existing (long) treatment durations. The RCTs allocate the participants to two arms of 2 × 59 episodes each: 3 vs. 6 weeks of targeted postsurgical systemic antibiotic therapy for implant-free SIs or 6 vs. 12 weeks for implant-related SIs. This equals a total of 236 adult SI episodes (randomization scheme 1:1) with a minimal follow-up of 12 months. All participants receive concomitant multidisciplinary surgical, re-educational, internist, and infectious disease care. We will perform three interim analyses that are evaluated, in a blinded analysis, by an independent study data monitoring committee. Besides the primary outcome of remission, we will also assess adverse events of antibiotic therapy, changes of the patient’s nutritional status, the influence of immune suppression, total costs, functional scores, and the timely evolution of the (surgical) wounds. We define *infection* as the presence of local signs of inflammation (pus, wound discharge, calor, and rubor) together with microbiological evidence of the same pathogen(s) in at least two intraoperative samples, and we define *remission* as the absence of clinical, laboratory, and/or radiological evidence of (former or new) infection.

**Discussion:**

Provided that there is adequate surgical debridement, both RCTs will potentially enable prescription of less antibiotics during the therapy of SI, with potentially less adverse events and reduced overall costs.

**Trial registration:**

ClinicalTrials.gov, NCT04048304. Registered on 5 August 2019.

**Protocol version:**

2, 5 July 2019.

## Background

Surgical site infections are feared complications of spinal surgery, the volume of which is expected to increase every year worldwide [1]. Likewise, community-acquired spinal infections (SIs) are associated with increased morbidity and costs and prolonged hospital stay for the patients [1]. Most scientific papers are interested in the epidemiology of SI and risk factors for surgical site infections after spinal surgery [2], occurring at 1–3% [2–4], rather than the modalities and outcomes of treatment. Risk factors leading to infection may be multiple. To cite an example, according to our University Spine Center at the Balgrist University Hospital in Zurich, associated risk factors are a high serum creatinine level, blood loss, or steroid use. Some of these risk factors influence the occurrence of infection only indirectly and act as a confounding element. For example, fusion surgery, particularly if it involves the lumbosacral spine, and length of surgery are associated with high blood loss [2], which itself may become an independent risk factor for infection. This remains the domain of infection control.

In contrast, we are interested if we can streamline antibiotic therapy after the occurrence of infection, especially by shortening its duration. Such results can be of high value for clinicians. So far, the literature on antibiotic regimens in SI is very sparse and strongly eminence-based (instead of being based on evidence). Most experts recommend a minimum (intravenous) antibiotic course duration of 2–4 weeks, often followed by prolonged oral antimicrobial regimens in case of infected osteosynthesis material that was kept in place [1]. Comparative data supporting these individual therapeutic recommendations are lacking. Indeed, one coauthor of the current project analyzed long-term remission with an emphasis on surgical and antibiotic-related parameters. The patients had a median of two surgical debridements with a median duration of antibiotic therapy of 8 weeks, during which the therapy was delivered parenterally for 2 weeks. In 53 cases (80%), the episodes were in complete remission. In cluster-controlled multivariate Cox regression analysis adjusting for the case mix, the duration of postsurgical antibiotic therapy was completely indecisive regarding the “remission of infection” or “mechanical sequelae” [1]. Especially, the following clinically important variables were all unrelated to remission: number of surgical interventions (hazard ratio [HR], 0.9; 95% confidence interval, 0.8–1.1), infection due to *Staphylococcus aureus* (HR, 0.9; 0.8–1.1), infection due to local antibiotic therapy (HR, 1.2; 0.6–2.4), and duration of total (HR, 1.0; 0.99–1.01) or just parenteral (HR, 1.0; 0.99–1.01) antibiotic use [1].

If there is no benefit to long duration antibiotic therapy, it would be important to limit the use of antibiotic agents to avoid furthering the problem of antibiotic resistance and adverse events, because the incidence of adverse events related to antibiotic therapy (substantial adverse events in up to 29% of all treatment episodes [5]) and costs genuinely increase with longer duration of antimicrobial administration [5]. We equally think that as long as oral antibiotics are used with good bioavailability and bone tissue diffusion, the antimicrobial treatment can be considerably shortened for the benefit of patients and the healthcare sector [6].

## Methods

### Setting

The Balgrist University Hospital (incorporating the University Spine Center Zürich) is a tertiary referral center for SI that is affiliated with the University of Zurich, Switzerland. Regarding SIs, it has a multidisciplinary team composed of five spine surgeons (both orthopedic and neurosurgery), three internist physicians, a hospital pharmacist, specialized wound nurses, musculoskeletal expert radiologists, three specialized nutritionist nurses, two to four dedicated physiotherapists, and up to four infectious disease physicians who specialize in orthopedic infections. Moreover, this team is supported by a research campus (Balgrist campus) with biobanking facilities and a Unit for Clinical and Applied Research with nine study nurses and two personnel with experience in biostatistics and investigational designs (www.balgrist.ch). Our study starts at Balgrist but is expandable to other national or international centers with experience in the treatment of SIs.

### Study objectives

We plan a prospective randomized study of SIs for which intraoperative debridement is part of the therapy. The primary study objective is to evaluate if 6 weeks of systemic and targeted antibiotic therapy postoperatively is not inferior to 12 weeks (noninferiority trial) in cases of infection in spinal implant-associated infections when spine implants are left in place. For SIs without implants, this objective is the evaluation of whether 3 weeks of antibiotic therapy is not inferior to 6 weeks in postoperative SIs without an implant. The switch from intravenous to oral medication will occur early, in the absence of sepsis *sensu strictu*, bacteremia, or intestinal problems, at the latest after 1 week of treatment. Secondary objectives are assessments of differences in total costs, sick leave, adverse events, mechanical sequelae, handicap at 6 and 12 months after treatment, and changes in nutritional status during therapy. A third objective is the assessment of infected tissue/bone for future studies (Biobanking).

Finally, our study includes the evaluation of the nutritional status of the patient at the beginning and the end of SI treatment. Instead of throwing tissue/bone away, we will collect intraoperative tissue and/or vertebral bone for other studies. Of note, biobanking and participation in the clinical trial are exclusive of each other. Patients refusing to provide intraoperative tissue for biobanking still have the choice to participate in the randomized study and vice versa.

### Definitions and eligibility criteria for participants

**Fig. 1:**
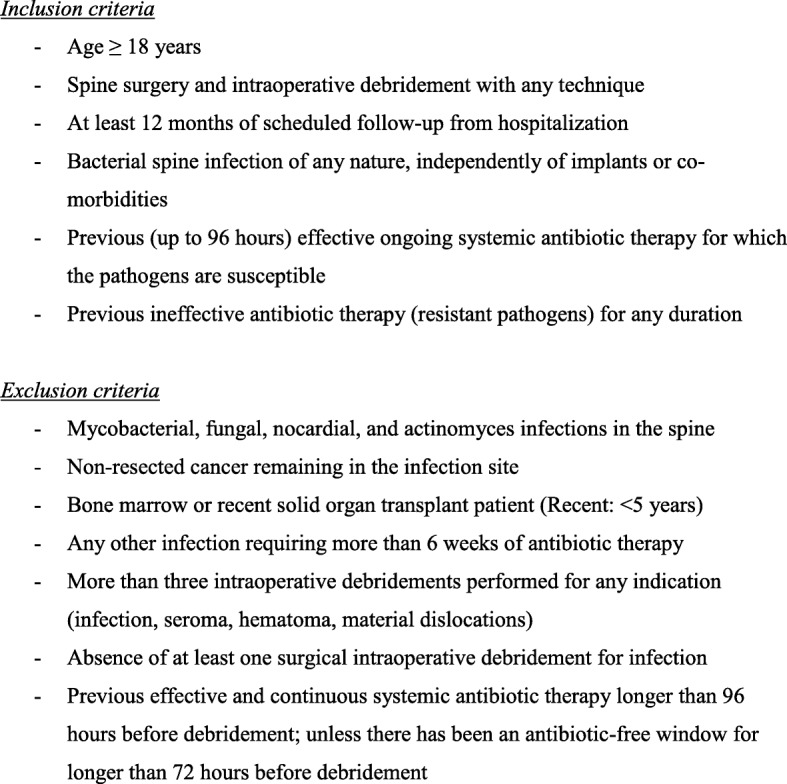
Study criteria

**Fig. 2:**
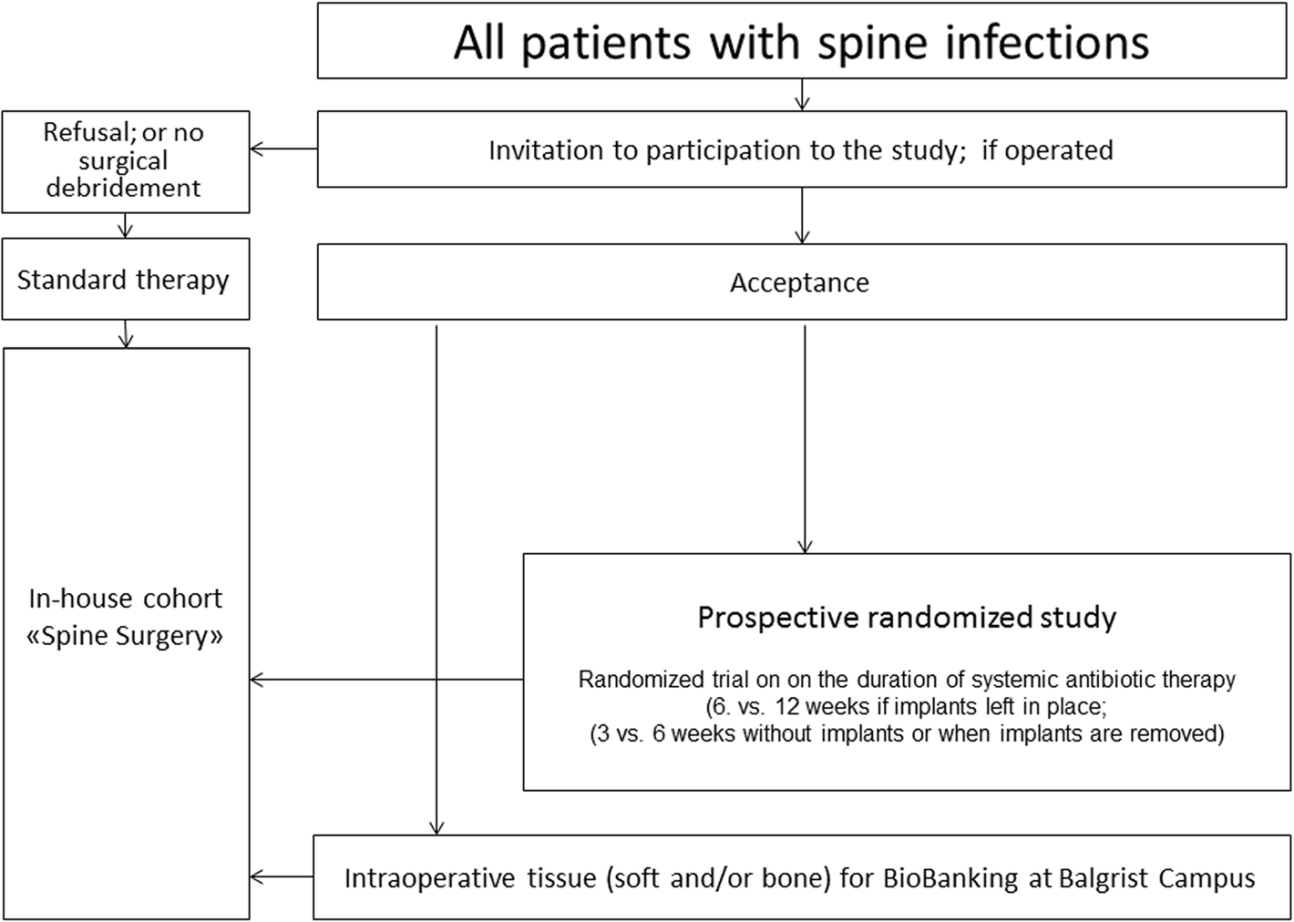
Study flowchart

SI is defined as having at least two local manifestations of inflammation (swelling or induration, erythema, local tenderness or pain, local warmth, purulent discharge) together with the same pathogen(s) retrieved in the microbiological culture of at least two intraoperative samples in antibiotic-naive cases. Systemic inflammation (fever, shivering, bacteremia, hemodynamic alterations) or histological confirmation is considered facultative. Remission is defined as the absence of any clinical, anamnestic, radiological, or laboratory signs of former (or new) SI during 12 months of follow-up. A diagnostic control puncture for the microbiological exclusion of dormant bacteria is not necessary. Of note, internal closed fractures and residual back pain can be interpreted as remission as long there are no signs of infection as defined. Figure 1 displays the inclusion/exclusion criteria, and Fig. 2 shows the study flowchart.

### Interventions and study conduct

Upon individual consent of the patient, we will collect clinical, radiological, nutritional, and laboratory data from each SI episode. The biobank will store intraoperative specimens in the Balgrist campus for 10 years. Table 1 reveals the variables of interest that we will collect during the trials. The two randomized controlled trials (RCTs) depend on the presence or absence of infected osteosynthesis material:

- *Infected spine material that was not entirely removed (or new material inserted):* Randomization between 6 and 12 weeks (± 4 days) of total antibiotic therapy counted since the first debridement for infection; early switch to oral targeted therapy
- *Infected spine without residual material:* Randomization between 3 and 6 weeks (± 4 days) of total antibiotic therapy counted since the first debridement for infection; early switch to oral targeted therapy

**Table 1:** Prospectively assessed variables

***Patients’ general descriptive characteristics:*** age, sex, body mass index, comorbidities, anticoagulation, known immunosuppression (diabetes mellitus, renal dialysis, cirrhosis, pregnancy, iatrogenic immunosuppression, untreated human immunodeficiency virus [HIV] infection, agranulocytosis, active cancer), American Society of Anesthesiologists (ASA) physical status classification, Nutritional Risk Screening (NRS 2002), neck disability index (NDI), or Oswestry low back disability index (ODI)	
***Patients’ spine surgery-specific baseline data:*** number and type of surgeries for the actual problem; agent, dose, and duration of presurgical antibiotic therapy; agent and duration of perioperative prophylaxis during debridement; cell count (absolute number and percentage of leukocytes); initial serum C-reactive protein (CRP) level; presence of initial bacteremia; presence of vertebral osteomyelitis; and presence and type spinal implants. Spine surgery data: anatomical localization of surgery, type of surgery, microbiological results, and histology (*if applicable*)	
***Treatment and outcome:*** number of surgeries to treat infection; total duration of antibiotic therapy; duration, agent, and dose of intravenous and oral antibiotic therapy; intraoperative vancomycin powder; wound-healing problems; presence and duration of vacuum-assisted negative pressure therapy; adverse events; clinical or and microbiological recurrence; date and reasons for rehospitalization and retreatment; follow-up data; fatalities; Nutritional Risk Screening (NRS 2002); neck disability index (NDI); or Oswestry low back disability index (ODI)	
***Administrative data:*** total hospitalization and outpatient costs, duration of hospital stay, duration of sick leave, duration of inpatient rehabilitation	
***One or two intraoperative bone and soft tissue samples*** for the biobank at Balgrist campus	

**Fig. 3:**
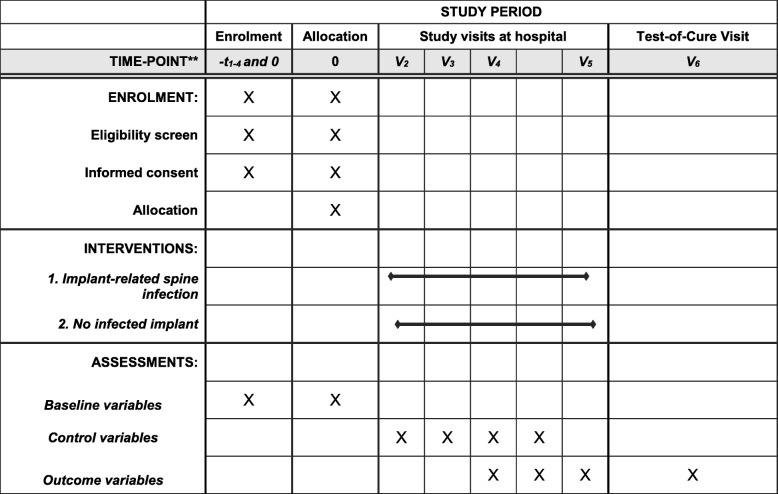
Standard Protocol Items: Recommendations for Interventional Trials (SPIRIT) chart of the enrollments and assessments during both randomized controlled trials

After randomization, the study participants will be actively followed for 12 months. At database closure, we will review the medical charts of all patients to seek unscheduled visits since inclusion. This “passive follow-up” can reach up to 4 years and terminates at the date of database closure. The scheduled study visits take place as follows: visit 1, enrollment (day 1); visit 2, day 15 (± 5 days); visit 3, day 21 (± 5 days); visit 4, day 42 (± 5 days); visit 5, day 84 (± 5 days); end of treatment visit 6, day 21, 42, or 84 (± 5 days) (only if still receiving treatment after visit 4); test-of-cure visit, approximately (± 60 days) at 12 months (visit 7). The Standard Protocol Items: Recommendations for Interventional Trials (SPIRIT) diagram in Fig. 3 shows the timely assessments that are identical for both RCTs.

### Antibiotic agents

The antibiotic therapy is prescribed by infectious disease physicians with experience in orthopedic infections, the surgeons in charge of the patient, and/or the internists. It is administered by nurses experienced in orthopedic infections. Initially, antibiotic therapy is either empiric or targeted to the results of preoperative bone biopsy. After 2–5 days, antibiotic therapy becomes targeted to the pathogens identified in microbiological cultures and their antibiotic susceptibility profile. The choice of the agent and its intravenous or oral administration route are usually at the discretion of the infectious disease physician. However, for this study, and in order to achieve minimal homogeneity, we established a list of “allowed antibiotics” and their recommended doses (Table 2). The investigators must choose from among them, unless the causing pathogen of the SI is not listed in Table 1 or if an additional surgical site infection (e.g., postoperative pneumonia) necessitates a broad-spectrum antibiotic treatment. Of note, in this study, we will not test special doses or new indications for antibiotic therapy. Only the duration of the therapy will be determined. All antibiotics are already on the Swiss market and approved by Swissmedic, the corresponding authority for medication use. We avoid placebos, topical antibiotics, and topical antiseptics, except for the preincisional skin preparation and (potential) use. Anesthesiologists and surgeons are also free to comply with the prevention protocols, even if the patient is already infected, by administering the standard antibiotic prophylaxis (cefuroxime, vancomycin, or clindamycin) for up to three consecutive doses.

**Table 2:** List of allowed antibiotic treatments (empirical or targeted)

Antibiotic agent	Allowed dosing regimens	Allowed total daily dose^a^
Levofloxacin by mouth	500 mg every 12 h	750 to 1000 mg
Ciprofloxacin by mouth	500 mg every 12 h	750 to 1500 mg
Amoxicillin/clavulanate by mouth	500/125 mg every 12 h or every 8 h	1000/250 mg to 1500/375 mg
Amoxicillin/clavulanate intravenous	1000/200 mg every 12 h or every 8 h	2000/400 mg to 3000/600 mg
Cefuroxime intravenous	1500 mg every 8 h	4500 mg
Ceftriaxone intravenous	2000 mg every 24 h	2000 mg
Co-trimoxazole by mouth	960 mg every 12 h or every 8 h	1920 to 2880 mg
Clindamycin by mouth	300 or 450 mg every 6 h	1200 to 1800 mg
Doxycycline by mouth	100 mg every 12 h	200 mg
Linezolid by mouth	600 mg every 12 h	1200 mg
Linezolid intravenous	600 mg every 12 h	1200 mg
Metronidazole by mouth	500 mg every 8 h or 500 mg every 6 h	1200 to 2000 mg
Metronidazole intravenous	500 mg every 8 h or every 6 h	1500 to 2000 mg
Vancomycin intravenous	15 mg/kg every 12 h	Target serum levels, 10–20 mg/L
Meropenem intravenous	1 or 2 g every 12 h or every 8 h	2 to 6 g
Piperacillin/tazobactam intravenous	4000/500 mg every 8 h	1200/1500 mg (12 g/1.5 g)

^a^To be adapted to renal insufficiency

#### Pregnancy and breastfeeding

This cohort, including all antibiotics and surgeries, has no specific relationship to pregnant or breastfeeding women and their children. Additionally, the study population is likely not to include women of childbearing age. Thus, pregnant and breastfeeding women are not excluded. The investigators will avoid agents that are not allowed for pregnant or breastfeeding women according to the Swiss Compendium (www.compendium.ch).

### Outcomes of interest

For the RCTs and biobanking, we will collect data and biological material. Concerning the RCTs, Table 1 (bottom) summarizes the outcome parameters. Regarding the investigation of dynamic changes in nutritional status during SI care, specialist nutrition nurses will assess the status at baseline and the end of treatment. In case of severe malnutrition, they are allowed to propose corrective measures already during the SI therapy because it would be unethical not to intervene only for study purposes. Finally, the database will be sufficiently large to estimate the influence of an underlying chronic immune suppression (i.e., diabetes mellitus, chronic steroid therapy, dialysis, untreated human immunodeficiency virus, active cancer in therapeutic or palliative treatment, CHILD C) on SI outcomes and related nutritional status. We also remind the reader that patients with very severe iatrogenic immune suppression, such as recent solid organ or bone transplant in the last 5 years, are exempted from SASI (Short Antibiotics for Spine Infections) trials (Fig. 1).

### Allocation and timetable

After written informed consent forms are given to participants (until day 5 of debridement), the unblinded allocation occurs electronically with a randomization scheme of 1:1 (randomization without blocked or matched variables). The study nurse of the Unit for Clinical and Applied Research and/or the coinvestigators will implement the allocation sequence in the trial. For both RCTs, we need 36 months of study time, starting from August 2019. Table 3 highlights some key time point events.

**Table 3:** Timetable of the study

Activity	2019				2020				2021				2022			
	P	S	A	W	P	S	A	W	P	S	A	W	P	S	A	W
Permission from ethics committees																
Ongoing recruitment of new sites																
Clinical study																
Database																
Interim statistical analysis																
Final statistical analyses																
Writing up of results and manuscript																

*Abbreviations: P* spring, *S* summer, *A* autumn, *W* winter

### Statistical analyses and sample size

Both RCTs are noninferiority trials. Remission incidence (at the first attempt of therapy) is set at 5% (5% recurrence in both arms). The maximum clinically acceptable difference (unidirectional noninferiority margin with binary outcome categorical variables) is arbitrarily fixed at 10% regarding the primary outcome of remission [1]. Assuming a risk of α = 0.05 and a power of 80%, it will be necessary to recruit 59 patients in each antibiotic duration arm (short or long). Together with the distinction of the RCTs as studies of implant-related and implant-free SIs, we will finally need 2 × 2 × 59 episodes, equaling a total of 236 SI episodes within 3 years. For assessment of the formal noninferiority requirement (regarding the primary outcome of remission), we will compute with a unidirectional *P* value limit of 0.025. We do not predefine a noninferiority margin for secondary outcomes such as costs, adverse events, functional outcomes, underlying immune suppression, dynamic changes in the nutrition status, and biobanking.

#### Interim analyses

When the first 20 episodes of any randomization branch will have complete follow-up, and again at 60 and 120 SI episodes, we will perform three interim analyses. On this occasion, we will equally check if the expected statistical power for the final analysis will be acceptable. If it is less than 30%, we will consider that the trial will not be able to demonstrate the result, and the recruitment will no longer be ethical. The most frequent conditional power evaluated under the current trend (i.e., using the information from the collected data) will be assessed [7, 8]. The study data monitoring committee will consist of independent surgeons or physicians with clinical and statistical experience who are not participating in the study. They will decide about the future of the trial, entirely or partially, after each of the three interim analyses. The principal investigator (PI) and the sponsor will present the data in a blinded form to the data monitoring committee. The committee members will only know if there is an implant, but they will ignore allocations to the antibiotic arms.

The intention-to-treat (ITT) population will consist of all randomized patients who have signed the consent for the participation. Patients will be analyzed according to treatment group assignment, regardless of whether they receive any treatment or the wrong treatment or are lost to follow-up. The per-protocol (PP) population will consist of all patients who complete the study and who have not deviated significantly from the protocol. The statistical analyses will mostly be based on descriptive analyses; group comparisons; and a multivariate, unmatched, eventually cluster-controlled Cox regression analysis adjusting for the large case mix that we expect. Equally, a generalized estimating equation model might adjust for clustering in case of multicenter origin of the patients. The biostatistician will analyze the datasets in a blinded form (as group A or B), but the PI, the study nurses, and the sponsor will ultimately unblind the allocations for data verification and definition of the ITT and PP populations.

### Ethical and regulatory aspects

#### Study registration, ethical conduct, and categorization

The study is approved by the Ethical Committee of Zurich (no. 2019-00646) and registered in the Swiss Federal Complementary Database (portal) and in the ClinicalTrials.gov international trial registry (NCT04048304). This study only makes use of the medicinal products and antibiotic agents that are already authorized in Switzerland. The indication and the dosage are used in accordance with the prescribing information and the international guidelines, making this study fall into the category of Clinical Trials A. The study will be carried out in accordance to the protocol and with principles enunciated in the Helsinki declaration, good clinical practice guidelines, and Swiss law. The ethical committee receives annual safety reports and is informed about the study stop/end. Substantial amendments are only implemented after a new ethical committee approval.

#### Patient information and informed consent

Participants will be recruited by any of the investigators of the study. Our institution has a standardized procedure for recruiting participants because participant studies are common. Each participant will be informed that participation in the study is completely voluntary and that he/she may withdraw from the study at any time and that withdrawal of consent will not affect his/her medical assistance and treatment in the future. All participants of the study will be provided a participant information sheet and informed consent form entailing sufficient information. For the biobank, the participants will sign the general consent for the further use of personal data and biologic material. The investigators affirm and uphold the principle of the participant’s right to privacy and that they shall comply with applicable privacy laws and/or the corresponding section of the study-specific consent.

### Safety issues

#### Monitoring

The Unit for Clinical and Applied Research of Balgrist University Hospital will assign an independent monitor. Regular monitoring visits at the investigator’s site prior to the start and twice during the course of the study will help us to follow the participants’ progress, to assure utmost accuracy of the data, and to detect possible errors at an early time point. The monitor will review all or a part of the case report forms (CRF) and written informed consent forms. The accuracy of the data will be verified by reviewing the above-referenced documents. There will be a close-out visit at the study end. During the monitoring, all documents, including source data/documents, will be accessible to the monitor.

#### Audits and inspections

An audit/inspection of this study may be conducted by the competent authority. The quality assurance auditor/inspector can have access to all medical records, the investigator’s study-related files and correspondence, and the informed consent documentation that is relevant to this clinical study. The investigator will allow the persons responsible for the audit or the inspection to have access to the source data/documents and to answer any questions that arise. All involved parties will keep the patient data strictly confidential.

#### Early termination of the study (participation)

The investigators may terminate the study prematurely according to certain circumstances, such as for ethical concerns, insufficient participant recruitment, when the safety of the participants is doubtful or at risk, alterations in accepted clinical practice that make the continuation of a clinical trial unwise, and early evidence of benefit or harm of the experimental intervention. If a patient is withdrawn, the reason will be noted. When possible, evaluations required at the next scheduled visit will be performed at early termination.

#### Treatment by specialists

All surgeries will be performed with the supervision and participation of an experienced spine surgeon. The antibiotic therapy is ordered and supervised by internists and infectious disease physicians with therapeutic and academic experience in SI treatments. The current medications of the study patients, as well as possible interactions, will be controlled by the internists several times per week during hospitalization.

#### Definition and assessment of (serious) adverse events and other safety-related events

An adverse event is any untoward medical occurrence in a patient and does not necessarily have a causal relationship with the study procedure. A serious adverse event (SAE) is classified as any untoward medical occurrence that results in death, is life-threatening, requires inpatient hospitalization or prolongation of existing hospitalization, and/or results in persistent or significant disability/incapacity. In addition, important medical events that may not be immediately life-threatening or result in death but may jeopardize the patient or may require intervention to prevent one of the other outcomes listed above should also usually be considered serious. Participants with ongoing SAEs at study termination will be further followed up until recovery or until stabilization of the disease after termination. The investigators will make a causality assessment of the event for the study. All SAEs must be reported immediately and within a maximum of 24 h to the sponsor-investigator of the study. SAEs resulting in death are reported to the local ethics committee (via local investigator) within 7 days. Patients who have experienced adverse events and are leaving the study will be treated off-study, without restriction, at the study site.

#### Case report forms, procedure of data analysis, and biobank archiving

An electronic CRF will be generated for every patient. All relevant study data are recorded by authorized persons using the REDCap® electronic data capture tool [9] and archived for a minimum of 10 years. Participating patients will be registered in an enrollment log assigning the participant to his/her study identifier. Corrections can be made only by authorized persons. For data analysis, subject-related data from REDCap will be exported and analyzed in statistical software (IBM SPSS Statistics and/or Stata). Before data export, all patient identifiers will be removed. Patient source and biobank data will be registered using subject identifiers. Collection, disclosure, and storage of patient-related data are carried out in accordance with Swiss data protection regulations and the Human Research Act. The biobank will store the intraoperative tissue samples in accordance with its guidelines (in RNALater at below-zero temperature (-20°C) in the Balgrist Campus. Likewise, radiological data are stored in the picture archiving and communication system according to the standard at the Balgrist University Hospital.

#### Theoretical risk of the study

Besides the retrospective identification of patients, we do not see any particular risk for the patients regarding the cohort. For biobanking specifically, a theoretical additional risk could be the detection of unknown pathologies if there would be further workup of the intraoperative samples. Concerning the RCTs, a theoretical risk could be a higher incidence of recurrences in the corresponding short-term antibiotic arms.

## Discussion

With our cohort in two embedded RCTs, we seek to demonstrate clinically relevant noninferiority of a shorter systemic antibiotic treatment in adult patients with SI with and without implants [1] and, independently of the surgical drainage technique, the number of debridements, underlying individual chronic immune suppression, the infection localization, or the pathogens. Importantly, all study participants will have accompanying multidisciplinary surgical, re-educational, internist, and infectious disease treatment and follow-up. We will equally collect intraoperative soft tissues and bone for future (laboratory) studies and assess adverse events, overall costs, functional outcomes, and the dynamic changes in nutritional status of the infected patients in relation to their therapy and outcomes. The studies will start in Zurich but are expandable to other study centers with experience in treating SI.

The primary outcome is remission at the last follow-up, but the RCT can be adjusted for different important variables, such as the number of surgical debridements, the use of a negative pressure therapy, administration of a parenteral antibiotic regimen, or the total duration of antibiotic therapy. As in many fields of septic orthopedic surgery, the number of surgical debridements does not formally influence remission rates, which has been shown for chronic osteomyelitis [10], septic native joint arthritis [11], fracture device infections [12], infected open fractures [11], or prosthetic joint infections [13]. There is very little evidence to guide surgical treatment of patients who require a single versus multiple debridements. Dipaola et al. [14] developed a predictive model for spinal surgical site infections based on 128 infected patients. Among 30 clinical variables analyzed, and despite the retrospective nature of their analysis, they validated 4 variables as being strongly predictive regarding the necessity of multiple debridements: infection due to methicillin-resistant *Staphylococcus aureus*, bacteremic disease, posterior lumbar spine, and use of nonautologous bone grafts.

Certainly, the most important variables retrieved from our trials will be antibiotic-related. Most author groups advocate a minimum length of parenteral antibiotic course of 2–4 weeks and a total duration up to 3 months [15, 16] for SIs, although some groups recommend only 2 weeks of parental therapy [17, 18], or even only 2–3 days [19], without further compromising success. To cite examples, Clark and Shufflebarger [20] treated delayed infections with surgery and 48–72 h of parenteral antibiotics followed by 10 days of targeted oral antibiotics. All infections were eradicated. Likewise, Richards and Emara [21] prescribed systemic antimicrobials only for 3 weeks, administered 2–5 days parenterally, followed by a 7 to 14 day-course of oral treatment.

In the entire field of orthopedic infections, there are no formal scientific data proving the benefit of a systemic antibiotic therapy beyond 6 weeks compared with 4–6 weeks or even less. Exceptions are by nature expert opinions in previous book chapters or past publications without their own database analyses or the therapy of special microorganisms requiring long-lasting antibiotic therapies such as mycobacteria [22], *Nocardia* spp. [23], and *Actinomyces* or fungi [24]. To cite recent and our own examples of investigations regarding the overall antibiotic duration, sacral osteomyelitis [25], long-bone osteomyelitis [10], fracture device-related infections [12], spondylodiscitis [25], prosthetic joint infections [13], diabetic foot osteomyelitis [26, 27], and many more failed to enhance remission rates if antibiotics were prolonged beyond 4–6 weeks, even in the presence of an infected implant. These emerging and relatively short durations are equally acknowledged by international consensus meetings [28] of surgeons and infectious disease physicians who treat these infections and perform research on them.

There are also studies with less than 6 weeks of total antimicrobial therapy, especially in the pediatric literature on hematogenous osteomyelitis. In this particular setting, a 3-week antibiotic course appears to be sufficient, as highlighted by many authors [29–32]. Among adults, 38 case series with antibiotic treatment durations of 3–4 weeks, including 5 to 36 patients each, revealed cure rates of approximately 80% according to a review published in 2005 [33].

A second issue is the distinction between intravenous and oral antibiotic administration, at least initially. Current textbooks recommend the parenteral route for at least the first 2 weeks for all osteoarticular infections [1, 34–36], but this recommendation is not evidence-based either. There are no predictive clinical markers that would justify prolonged initial intravenous administration. In addition, up to one-third of patients with chronic bone and implant infections may experience antibiotic-related or catheter-related problems during parenteral treatment [36]. For economic reasons as well as patient and nurse comfort, parenteral administration should be kept to a minimum [37]. Good bone penetration during parenteral and oral administration has been proven in several reports [38–40], and data suggest that an early switch to oral antibiotics is as effective as prolonged parenteral regimens [41].

A Cochrane review investigated five trials comparing oral vs. parenteral antibiotics in osteomyelitis. There was no statistically significant difference between the two groups in the remission rate 12 months or more after treatment [42]. Glassman et al. successfully treated two patients with SIs from the start with oral ciprofloxacin, an antibiotic with excellent oral bioavailability and bone penetration [43]. Even in cases of diabetic foot osteomyelitis, a frequent disease with a hallmark of vascular insufficiency and tissue ischemia, there are no data indicating the superiority of any particular route of delivery of systemic antibiotics [44]. Byren et al. demonstrated that an intravenous course of antibiotics for over 4 weeks did not enhance cure for the treatment of arthroplasty infections [45]. Zimmerli et al. summarized observational studies that showed the same failure rates of arthroplasty infection treatment despite a prolonged period (4–6 weeks) of intravenous treatment [46]. For the treatment of bone infections, there are some antibiotics that have already been proven to be effective in oral form. Quinolones, rifampicin, co-trimoxazole, tetracycline, or clindamycin have such a good and sufficient oral bioavailability [47].

Our future patient population will comprise all comorbidities and chronic immune suppressions. For example, we expect 20–25% of patients will have diabetes [48] in our center, along with other immune suppressions such as cancer, advanced cirrhosis, and steroid medication. While immune suppression (especially diabetes mellitus) is an acknowledged independent risk associated with healthcare-associated surgical site infections [49], its influence on remission during therapy for SIs is unknown. Indeed, all current therapeutic concepts for osteoarticular infections in general do not rely on the presence or absence of immune suppression [1, 10, 12, 25], suggesting that surgical debridement and long antibiotic administration overcome eventual shortcomings of patients’ immunity. Although our SASI trials do not target the association of immune suppression with SI outcomes, we will see if immune suppression tends to decrease remission when we shorten the antibiotic duration.

Finally, our RCT will also give insight into the nutritional status of patients with SI. Current literature is divided between experts advocating a causal relationship between malnutrition and occurrence of surgical site infections in orthopedic surgery and others who have retrospectively investigated this relationship and mostly found no associations [50]. Both factions know even less about the associations and the dynamics of nutritional status in already-infected orthopedic spine patients and the association of these alterations with remission, functional outcomes, and underlying immune suppression, let alone the question of the benefits of nutritional interventions during the combined surgical, physiotherapeutic, and antibiotic treatment [51]. This will be *terra nova* that we embed into our trials.

We do not expect major difficulties in performing our studies. Despite two prospective randomized designs (for SI with and without implants) and only 236 different episodes anticipated, patients’ voluntary participation might be low. Likewise, patients who continue to be treated outside of our center may be lost to follow-up or may have their treatment changed because the follow-up physicians do not agree. However, our center is the largest public hospital for surgical SIs in the region, and it is a university spine center, so this is unlikely to be a major bias. Last, and formally, our study participants will benefit from an initializing surgical debridement of infections. Hence, our results will not be valid for the conservative treatment of SI, which must not be confounded.

## Data Availability

The datasets used and/or analyzed during the current study are available from the corresponding author on reasonable request.
